# Beta blocker use in traumatic brain injury based on the high-sensitive troponin status (BBTBBT): methodology and protocol implementation of a double-blind randomized controlled clinical trial

**DOI:** 10.1186/s13063-021-05872-8

**Published:** 2021-12-07

**Authors:** Ayman El-Menyar, Mohammad Asim, Ahmed Abdel-Aziz Bahey, Talat Chughtai, Abdulnasser Alyafai, Husham Abdelrahman, Sandro Rizoli, Ruben Peralta, Hassan Al-Thani

**Affiliations:** 1 Clinical Research, Trauma & Vascular Surgery Section, Hamad General Hospital (HGH), PO Box 3050, Doha, Qatar; 2 Clinical Medicine, Weill Cornell Medical College, Doha, Qatar; 3 Department of Surgery, Trauma Surgery Section, HGH, Doha, Qatar; 4 Department of Surgery, Qatar University, Doha, Qatar; 5 Department of Surgery, Neurosurgery, HGH, Doha, Qatar

**Keywords:** Traumatic brain injury, Beta blocker, High-sensitive troponin, Randomized control trial, Functional outcome

## Abstract

**Background:**

Beta-adrenergic receptor blockers (BB) play an important role in the protection of organs that are susceptible for secondary injury due to stress-induced adrenergic surge. However, the use of BB in traumatic brain injury (TBI) patients is not yet the standard of care which necessitates clear scientific evidence to be used. The BBTBBT study aims to determine whether early administration of propranolol based on the high-sensitive troponin T(HsTnT) status will improve the outcome of TBI patients. We hypothesized that early propranolol use is effective in reducing 10- and 30-day mortality in TBI patients. Secondary outcomes will include correlation between serum biomarkers (troponin, epinephrine, cytokines, enolase, S100 calcium binding protein B) and the severity of injury and the impact of BB use on the duration of hospital stay and functional status at a 3-month period.

**Methods:**

The BBTBBT study is a prospective, randomized, double-blinded, placebo-controlled three-arm trial of BB use in mild-to-severe TBI patients based on the HsTnT status. All enrolled patients will be tested for HsTnT at the first 4 and 6 h post-injury. Patients with positive HsTnT will receive BB if there is no contraindication (group 1). Patients with negative HsTnT will be randomized to receive either propranolol (group 2) or placebo (group 3). The time widow for receiving the study treatment is the first 24 h post-injury.

**Discussion:**

Early BB use may reduce the catecholamine storm and subsequently the cascade of immune and inflammatory changes associated with TBI. HsTnT could be a useful fast diagnostic and prognostic tool in TBI patients. This study will be of great clinical interest to improve survival and functional outcomes of TBI patients.

**Trial registration:**

ClinicalTrials.gov NCT04508244. Registered on 7 August 2020. Recruitment started on 29 December 2020 and is ongoing.

**Supplementary Information:**

The online version contains supplementary material available at 10.1186/s13063-021-05872-8.

## Introduction

Traumatic brain injury (TBI) accounts for up to 30% of all injury-related deaths [1]. It also poses a significant morbidity and economic burden worldwide [2, 3]. Although there are significant advances in the overall trauma care, the management of head injury remains with certain limitations. Data from the Qatar national trauma registry database showed that the incidence of TBI is approximately 25% of the total trauma admissions. There are few studies evaluated the clinical significance of elevated serum cardiac troponins after trauma [4–7]. Some of these studies showed that elevation of serum troponin level is reflecting the severity of the overall body injury and stress-induced adrenergic storm in the absence of obvious direct cardiac contusion [5, 6]. Furthermore, elevated troponins have been reported in acute non-traumatic head insults, including acute stroke (≈27%) and subarachnoid hemorrhage (≈20%) [4, 7]. Troponin release is heart-specific but not disease-specific; therefore, the precise mechanism of elevated troponin in acute non-cardiac disorders remains difficult to determine. Prior data have shown that elevated troponins are commonly seen in critically ill patients in the absence of obstructive coronary artery disease [7–14]. The non-coronary diseases patients recognized by high-sensitive troponin were found to have a 23% higher risk of mortality in the intensive care units as compared to patients without troponin elevation [15]. Moreover, the clinical relevance and prognostic value of elevated troponins levels are poorly explored in TBI and polytrauma patients. However, prior studies have attributed the troponin release in the latter conditions to the occurrence of myocardial injury secondary to the catecholamine and inflammatory mediators surge in these stressful situations [8–11, 16].

Earlier studies have focused primarily on the clinical relevance of the conventional troponin T (TnT) or I (TnI) in patients with critical illness but did not examine well the newer high-sensitive TnT (HsTnT) which has a higher sensitivity and shorter time to detect minor myocardial damage [9]. An earlier study reported a higher rate of mortality in patients with elevated serum troponin levels as compared to normal troponin in isolated TBI patients [17]. The high-sensitive troponin I was tested in patients with mild TBI, and authors [18] have reported a significant association between positive HsTnI and the abnormal head CT scan findings as compared to a negative CT scan in those with negative HsTnI. A recent meta-analysis [19] reported that among TBI patients, the effect of elevated troponin on mortality had a pooled odds ratio (OR) of 3.3, which is almost like that reported in non-traumatic brain injuries studies.

A recent study from our center revealed an all-cause mortality of 26% in TBI patients and the mortality rate was 4-fold higher in those who presented with elevated HsTnT levels [20]. In this study, positive HsTnT was reported in 65% of patients with moderate to severe TBI. Moreover, on multivariate regression analysis, positive HsTnT was an independent predictor of in-hospital mortality even after adjusting for any chest trauma [20]. Myocardial injury, defined as at least single cardiac troponin concentration above the 99th percentile upper reference limit, has been reported following TBI with a likely dose-response relationship with the severity of the head injury [21]. In patients with isolated TBI, a recent study found significant correlations between the on-admission TnI value and Glasgow Coma Scale (GCS), head Abbreviated Injury Scale (AIS), and Acute Physiology and Chronic Health Evaluation II score (APACHE II) [22].

Beta-adrenergic receptor blockers (BBs) may reduce the cascade of immune and inflammatory changes associated with injury as well as the heart rate and blood pressure. Blunting the beta-adrenergic sympathetic surge using BBs has been shown to reduce mortality post-trauma and post non-cardiac surgery in several studies [8, 16, 23–25]. Early administration of propranolol was found to be independently associated with lower mortality among patients with moderate to severe TBI (adjusted odds ratio, 0.25) and having a better functional outcome (GOS-E) at 6-month follow-up [25, 26].

### Study rationale

To date, no randomized controlled trial (RCT) has focused on the early administration of BBs in TBI patients based on the HsTnT status. BBs administration is still not the standard of care for TBI patients. However, prior observational studies have suggested that early low-dose propranolol can be safely administered and might improve outcomes after TBI [25, 27, 28] or severe trauma [29]. According to a recent meta-analysis [30], there is a single RCT that assessed the potential adverse events associated with BB therapy in TBI. There was no significant difference between the study groups in terms of the incidence of hypotension, bradycardia, heart failure, and bronchospasm.

The question of whether early administration of BBs has beneficial effect on the 10- and 30-day mortality in patients with mild-to-severe TBI based on the admission HsTnT status will be investigated in this study. Of note, there are various questions that need exploration such as (a) whether the severity of head injury alone or in combination with biomarkers will be a guide for the early use of BBs in TBI, (b) whether these biomarkers reflect the sympathetic surge only or the silent myocardial injury as well, and (c) whether these biomarkers have prognostic value only or they also possess diagnostic and therapeutic implications in TBI. To address this gap in the literature, we are undertaking a large double-blind RCT comparing protocolized, early administration of propranolol (within the first 24 h of hospital admission and after the HsTnT result) in the management of TBI patients. This study will be powered sufficiently to detect clinically relevant differences in all-cause mortality and neurological outcomes.

## Methods and study design

The Beta Blocker Use in Traumatic Brain Injury Based on the High-Sensitive Troponin T Status (BBTBBT) is a prospective double-blind RCT of BB use in TBI patients based on the HsTnT status. The primary hypothesis is that early propranolol administration is effective in reducing the10- and 30-day mortality.

### Participants and study setting

The trial setting is the Hamad level I Trauma Centre in the state of Qatar, and the study subjects will be screened and enrolled from the trauma room in the emergency department (ED). Inclusion criteria are adult patients (≥ 18 to ≤ 65 years old) who sustained mild-to-severe blunt TBI (head AIS 1-5 and/or GCS 4-15), screened and enrolled within the first 24 h post-trauma.

Exclusion criteria are non-survivable injuries (head AIS = 6 and GCS = 3), uncontrolled bleeding on arrival to ED, SBP ≤ 100 mmHg (or MAP < 70 mmHg) not responding to initial resuscitation or required to be maintained on vasopressors for persistent hypotension (up to 24 h), patients with bradycardia [HR < 60] (re-assess every 2 h), pregnant women, prisoners, known to have severe bronchial asthma, and who requires emergency surgery < 6 h and no longer under trauma team care.

**Fig. 1:**
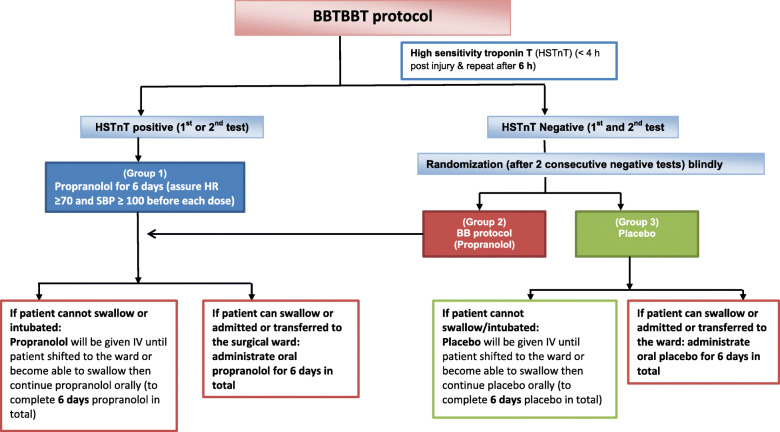
BBTBBT protocol

We expect to recruit a total of 771 TBI patients over 2 years. On hospital arrival, all TBI patients will have an initial head CT scan and managed according to the Advanced Trauma Life Support (ATLS) guidelines. We have a neurocritical care team with the state-of-art monitoring, imaging, and management of TBI. For this study, all mild-to-severe TBI patients who required hospital admission will be screened and enrolled by the attending physician and assigned nurse, if the subject meets the eligibility criteria. All enrolled patients will be tested for HsTnT (on admission and to be repeated after 6 h if the first reading is normal) and those who had positive HsTnT will receive BB, if no contraindication (group 1). Subjects with negative HsTnT (2 consecutive tests) will be randomized to either group 2, HsTnT –ve and BB use, or group 3, HsTnT –ve and no BB (placebo) (SPIRIT, Fig. 1).

Patients will be stratified based on the admission GCS prior to randomization. Serial electrocardiography (ECG) will be performed for all patients. Patients with abnormal ECG and/or positive troponin will also undergo transthoracic echocardiography, if indicated. Participant timeline is given in Table 1

**Table 1:** BBTBBT timeline

Procedure/intervention/task	Day 1	Day 2	Day 3	Day 4	Day 5	Day 6	*Day 10	*Day 30	3-month
Selection criteria^1^	X								
Informed consent	X								
CT scan head^2^	X								
HsTnT^3^	X								
Vital signs^4^	X	X	X	X	X	X			
Randomization	X								
Treatment protocol^5^	X	X	X	X	X	X			
Electrocardiography (ECG)	X	X							
Echocardiography^6^	X								
Laboratory tests (S100B, enolase, cytokines (IL-1b,6,8,18))	X	X	X						
Serum epinephrine	X	X							
Routine blood tests^7^	X								
Glasgow Outcome Score (GOS)									X
Last day follow-up									X

1 = on admission, 2 = on-admission if hemodynamically stable and head injury suspected, 3= within 4 h and to repeat after 6 h, 4= continuous monitoring and to be documented before starting treatment, 5 = beta blocker vs. placebo within the 1st 24 h of admission, 6 = if troponin positive and ECG changes,7 = blood hemoglobin, platelet count, international normalized ratio, prothrombin time, activated partial thromboplastin time, blood glucose level, C-reactive protein, serum creatinine, urea, lactate, base deficit, sodium, and potassium, * = outcome (alive vs. dead)

### Outcome measures

- The primary outcome is to determine whether early administration of propranolol based on the HsTnT status influences the 10- and 30-day mortality in TBI patients.
- Secondary *endpoints* include the effect of propranolol administration on the duration of hospital stay and functional status, measured using Functional Independence Measure (FIM) score at the latest time and Glasgow Outcome Score (GOS) at 3 months.
- We will also perform secondary exploratory analyses: (i) to correlate the HsTnT value with the serum S100 calcium binding protein B and enolase (markers of brain injury) value and the injury severity, (ii) to correlate the HsTnT value with the inflammatory markers and *e*pinephrine, and (iii) to examine the association between the HsTnT result and type of TBI lesion based on the CT scan.

#### Which BB will be used

Propranolol is an ideal BB agent as shown in some observational studies because of its non-selective inhibition and lipophilic properties, and thus, it has a potential to reduce secondary injury and stress after TBI [25, 31]. Moreover, propranolol is a cheap BB agent available in a generic form only and has plasma half-life of 2 h after its intravenous administration. It is completely absorbed after oral use with peak plasma concentrations occurs 1–2 h in a fasting patient [32]. The liver eliminates around 90% of the oral dose with an elimination half-life of 3 to 6 h. It is widely and rapidly distributed throughout the body especially in the lungs, liver, kidney, brain, and heart [32].

#### Propranolol administration protocol

The current study will examine the early use of propranolol which will be administered for the initial 6 days post-injury. The first dose of propranolol will be administered within the first 24 h of hospital admission at 1 mg intravenous every 8 h for 2 days, followed by 1 mg propranolol intravenously every 12 h for another 2 days and finally 1 mg propranolol will be administered intravenously at every 24 h for 2 days (oral propranolol will be given to patients who are alert and can swallow). Heart rate and blood pressure will be recorded before giving the BB/placebo dose and throughout the study period.

#### Imaging and laboratory investigations

Once head injury is suspected, serum HsTnT test will be done, and patient will be sent to have a head CT scan. ECG will be done on admission and patient will have an echocardiography if troponin test comes positive and/or abnormal ECG (i.e., ST-T changes). S100B, enolase, epinephrine, cytokines (IL-1b,Il-6,Il-8,and Il-18), blood hemoglobin, platelet count, international normalized ratio, prothrombin time, activated partial thromboplastin time, blood glucose level, C- reactive protein, serum creatinine, urea, lactate, base deficit, sodium, and potassium. Epinephrine, brain, and inflammatory markers tests will run in duplicates. HsTnT was assayed using Elecsys (Roche Diagnostics International Ltd.). Myocardial injury will be defined as HsTnT cut-offs of 14 ng/L for women and 22 ng/L for men or a single cut-off of 19 ng/L [33].

#### Strategies to improve adherence to intervention protocols

Regular educational meetings, auditing, and agreement with the medical staff and neurosurgeon will be followed on regular basis to address the logistics for drug administration, precautions, and potential side effect and its management.

#### Randomization

The total number of HsTnT negative TBI patients will be randomized into two groups on the basis of administration of BB or no BB (placebo) with an allocation ratio of 1:1. The random number sequence will be generated using a random number table (simple randomization) which will be sequentially numbered and sealed in opaque envelopes of block size of ten.

The “randomization with concealment” procedure will be used for the allocation of patients into different randomization groups. The random number sequence will be generated using the Robust Randomization App (RRApp) which is a publicly available resource to generate rigorous and reproducible randomization schemes. We will generate the random number sequence by the process of simple randomization with a block size of ten which will be sequentially numbered and kept in sealed opaque envelopes for treatment assignment. Furthermore, to minimize the selection bias, we will strictly maintain allocation concealment from those assigning participants to study groups, implementation of protocolized treatment for all study participants, and blinded assessment of the primary outcome by trained research staff unaware of the sequence. Subjects will be enrolled in the trial by the study team, and the randomization for BB use among the HsTnT-negative group will be on double-blind basis. Details will be documented on the wristband and label (patient’s initials, date and time of randomization). The physician/nurse will document the randomization of the patient on the case report form. The wristband and label in the patient’s medical record will alert hospital staff to the patient’s enrolment in the trial.

#### Blinding

Active and control study medications will appear identical. The treatment packs will be numbered, and the codes will be kept confidential and not broken until needed for emergency unblinding. The unique study ID number printed on the trial pack and the contents within the pack will be the only way to determine whether a pack contains active drug (propranolol) or placebo (0.9% normal saline).

#### Emergency unblinding

In the unlikely event of an emergency where the appropriate treatment of the patient requires knowledge of the study drug, the site investigator may unblind the treatment assignment by contacting the research coordinator assigned for emergency unblinding. The study code should only be broken when the treating physician consider that the clinical treatment relies crucially on whether the patient received BB or placebo.

#### Withholding (temporary cessation of BB protocol)

As per the BB protocol, vital signs such as HR and SBP will be specifically monitored before administration of the next dose of propranolol. The study therapy (active or control) will be withheld, if the HR drops ≤ 70 beat/min, SBP ≤ 100 mmHg (with no response to fluid resuscitation within 2 h), or MAP < 70 mmHg. Patients initiated on the BB protocol and in whom there is failure to continue the subsequent doses of propranolol as per the BB protocol will also remain in the study and will be followed as per protocol. Data from such cases will also be analyzed based on the intention-to-treat principle.

#### Study consent

There are 4 types of consent included in this study protocol. This study involves a time-critical intervention in TBI patients who may be unconscious, unable to consent to participation or no next of keen available within the therapeutic window of the study; therefore, a deferred consent will be obtained for subject’s enrollment in the study and signed by 2 physicians. In this case, the subject will receive the allocated study intervention provided by the treating independent physician and a study team member. Whenever available, the participant’s next-of-kin will be asked to sign consent to continue subject’s participation in the study. Patient who recovers enough cognition to understand the details of the consent will be asked to consent for continuation in the study (delayed informed consent). Patient, who presents with mild injury and can communicate well, will be asked to sign a written formed consent. In all the scenarios, a copy of the consent will be provided and refusal or withdrawal from the study at any time is the patient right and this will not affect his access for full standard of care management. The consent will allow the subject to permit/or decline the use of leftover blood samples to be used for further research.

### Data management

The Trauma Surgery research section at HGH will be responsible for project coordination and data management activity. A pre-designed paper case report form (CRF) will be used as source document to individually record all relevant information regarding the enrolled subjects. Prospective data collection including follow-up assessment (FIM and GOS) will be monitored for accuracy and consistency by the study team members on a regular basis. Complete data will be obtained for all study subjects, including those who fail to continue the BB protocol. The de-identified data from the individual CRF will be entered into a secure password protected computer. After study completion, the CRF, consent form, and data will be stored in accordance with the local regulatory requirements. The PI will provide specific code numbers to each specimen collected and only the project/data manager will know the subject identifier. The investigator will track all the specimens received by allotting unique subject identifier for all blood specimens and track the sample transportation, processing, and consumption. After study finishes, link between code and identifier will be destroyed. The de- identified data will be kept for a period of five years and then will be destroyed.

### Study end points and completion

We expect to complete the targeted subject recruitment and follow-up within 24 months. A subject will be in the research study until 3 months post enrolment or death. Patients will be followed up for Functional Independence Measure (FIM) at the latest time measured and Glasgow Outcome Score (GOS) at 3 months (in the clinic or through telephonic script). Patients and/or their next-of-kin will be asked to provide contact numbers to assess the outcome during follow-up.

### Statistical analysis plan

The proposed sample size is based on an estimated rate of 12% reduction in mortality with the use of BBs in TBI patients reported by Salim et al. [13], having 80% power and 5% alpha error; the estimated sample size would be 257 subjects (including 5% dropout) in each study group (total 771 patients).

All analysis will be blinded to treatment assignment and will be performed by an independent biostatistician at Hamad Medical Corporation. We will also perform multivariate logistic regression analysis after adjusting for relevant covariates in addition to the differential intervention with an adjusted risk ratio and 95% confidence interval for the probability of death. Analysis will be based on intension-to-treat. Additional exploratory analyses will be performed for continuous variables such as marker of brain injury, injury severity scoring and head Abbreviated Injury Scale, cytokine level by comparing their distributions between intervention arms, and then by multiple linear regression models including other potential covariates of interest. We will explore the effectiveness of the intervention in subgroups using stratified analysis as well as multivariable models. The response to BBs use based on the type of brain lesion will be analyzed. Data analysis will be carried out using the Statistical Package for Social Sciences version 26 (SPSS Inc. USA).

### Data and safety monitoring and interim analyses

An independent data safety monitoring board (DSMB) will monitor and assess the safety of the trial and review the serious adverse events across the study duration. The DSMB will comprise of experts in clinical trials, biostatistics and trauma care. The DSMB meetings will be held every 3 months. All adverse events will be reported to the institutional review board (IRB) and to the DSMB members. Unanticipated /fatal adverse events will be reported to the IRB within 24 h of the determination of unanticipated adverse event by the research team. The investigators will perform interim safety analyses at 12 and 24 months following 25% (*n* = 193) and 50% (*n* = 386) patient recruitment. The PI will keep IRB posted regarding DSMB review outcomes (PI will submit the minutes of the DSMB meeting report to IRB). All fatal events will be reported by the PI to the DSMB and IRB within 24 h of their incidence. If the mortality rate exceeds more than 50% in any therapeutic arm, the study team will stop the study and notify IRB.

### Anticipated results and clinical implications

This RCT will be of great clinical interest to improve survival and functional outcomes of TBI patients, as there are no guidelines or consensus for management of mild-to-severe TBI patients based on cardiac marker (i.e., HsTnT status) or the impact of stress. We believe that early administration of BBs (propranolol) has beneficial effect on the 10- and 30-day mortality in patients with mild-to-severe TBI. We anticipate better understanding of TBI that may lead to improved patient management, outcomes, and reducing the health care cost. Early BBs use reduces the catecholamine storm and subsequently the cascade of immune and inflammatory changes associated with TBI. HsTnT could be a useful fast diagnostic and prognostic tool in TBI patients. This study will be of great clinical interest to improve survival and functional outcomes of TBI patients.

#### Ancillary and post-trial care

Propranolol is a well-known FDA-approved drug since decades. It has short half-life with no long-term adverse events. It will be given under continuous monitoring of patient hemodynamics in a level 1 trauma center, and any potential side effect will be managed immediately and reported to the medical research center. All our trauma patients are receiving the state-of-art management free of charge including medications, rehabilitation, and long-term care.

#### Future directions and plan of action

Disseminating the results of our proposal (at local, regional and international level) will enhance prospects for further support and collaborative work. We will follow authorship eligibility guidelines and will not use professional writers. Dissemination of final report will include participation in scientific relevant congress and publication in peer-review journals. We are planning to seek multicenter collaboration for the current RCT, if feasible (so far, the current COVID pandemic limits this option). We anticipate that the important findings of this study will help improving the TBI management in our institution.

## Discussion

From the therapeutic point of view, BBs use was reported to have better survival in blunt TBI patients [4, 13, 14, 25, 26, 34–40]; however, double-blind RCTs remain lacking. Despite the optimistic results from prior data, a protocolized early BBs use is not currently a routine practice in patients sustaining TBI. Moreover, the release of cardiac troponin after head injury needs more scientific elaboration and physician awareness. A recent meta-analysis involving 12,721 patients showed that administration of BBs after TBI was associated with a significant reduction in adjusted in-hospital mortality (OR, 0.39) [35]. Retrospectively, Salim et al. [13] reported that patients with severe TBI who did not receive BBs had a mortality rate of 36% vs. 24% in those who received BBs (*p* = 0.036). Furthermore, if troponin-I was elevated on admission, the hospital mortality increased to 48.5% in patients without BBs therapy vs. 22.4% in those who received BBs (*p* = 0.026). In brief, the greatest survival benefit was observed retrospectively in the BBs group based on the conventional troponin positivity on admission.

Murry et al. [27] conducted a prospective observational study to administer low dose propranolol in 28 moderate-to-severe TBI patients. The authors suggested that early low-dose propranolol can be safely administered and might improve outcomes after TBI. A recent study by Ley et al. [39] observed a survival benefit in patients who received BBs before as well as after adjustment for injury severity. However, the administration of BBs was not protocolized, and there was no consistency in dosage, type, regime, and timing of BB administration [8]. In the DASH study (single center, randomized, double-blinded, placebo-controlled trial), one arm received propranolol and clonidine, and the other arm received double placebo, within 48 h of severe TBI [41]. The primary outcome was ventilator-free days. The study used two medications that could aggravate hypotension in TBI. Moreover, BB was not given early, but rather within 48 h post injury and mortality was not the primary outcome. In the BBTBBT, we are going to use propranolol vs. placebo in hemodynamically stable TBI patients and based on the HsTnT status. The propranolol will be given to all eligible patients with positive troponin but will be randomized only in those who have negative troponin. This study will protocolize the type, dose, route, and frequency of BB in TBI. It will also address which injury severity and brain lesion that could gain more benefit after BB use. It will provide better understanding of the role of troponin release after TBI and its correlation with brain biomarkers and cytokines.

## Trial status

The study started recruitment on 29 December 2020 and to be completed in 31 December 2022. The study was registered on 07 August 2020 at ClinicalTrials.gov (identifier number: NCT04508244).

Date and version identifier:

Protocol code: BBTBBT

Version: August 20; 2020

MRC, Hamad Medical Corporation: IRGC-05-SI-18-293

Amendment: 29 July 2020 and 23 February 2021 (the initial time widow to administrate BB was the first 12 h; it has been amended to be the first 24 h; this will ensure better hemodynamic stability before starting BB).

IRB-A-HMC-2019-0014

## Supplementary Information

**Additional file 1.**

## Abbreviations

RCT: Randomized clinical trial
BB: Beta blockers
TBI: Traumatic brain injury
HsTnT: High-sensitive troponin T

## References

[1] Faul M, Xu L, Wald MM, Coronado VG (2010). Traumatic brain injury in the United States: emergency department visits, hospitalizations, and deaths.

[2] Ma VY, Chan L, Carruthers KJ (2014). Incidence, prevalence, costs, and impact on disability of common conditions requiring rehabilitation in the United States: stroke, spinal cord injury, traumatic brain injury, multiple sclerosis, osteoarthritis, rheumatoid arthritis, limb loss, and back pain. Arch Phys Med Rehabil.

[3] Mock C, Lormand JD, Goosen J, Joshipura M, Peden M (2004). Guidelines for essential trauma care.

[4] Al-Otaiby MA, Al-Amri HS, Al-Moghairi AM (2011). The clinical significance of cardiac troponins in medical practice. J Saudi Heart Assoc.

[5] Mahmood I, El-Menyar A, Dabdoob W, Abdulrahman Y, Siddiqui T, Atique S, Arumugam SK, Latifi R, Al-Thani H (2016). Troponin T in patients with traumatic chest injuries with and without cardiac involvement: insights from an observational study. N Am J Med Sci.

[6] Edouard AR, Felten ML, Hebert JL, Cosson C, Martin L, Benhamou D (2004). Incidence and significance of cardiac troponin I release in severe trauma patients. Anesthesiology..

[7] Hasanin A, Kamal A, Amin S, Zakaria D, El Sayed R, Mahmoud K, Mukhtar A (2016). Incidence and outcome of cardiac injury in patients with severe head trauma. Scand J Trauma Resusc Emerg Med.

[8] El-Menyar A (2018). Beta blockers therapy in traumatic brain injury: is it the time to disclose the brain-cardiac interactions?. J Trauma Acute Care Surg.

[9] Freund Y, Chenevier-Gobeaux C, Bonnet P (2011). High-sensitivity versus conventional troponin in the emergency department for the diagnosis of acute myocardial infarction. Crit Care.

[10] Smith A, John M, Trout R, Davis E, Moningi S (2009). Elevated cardiac troponins in sepsis: what do they signify?. W V Med J.

[11] Lim W, Cook DJ, Griffith LE, Crowther MA, Devereaux PJ (2006). Elevated cardiac troponin levels in critically ill patients: prevalence, incidence, and outcome. Am J Crit Care.

[12] Poe S, Vandivier-Pletsch RH, Clay M, Wong HR, Haynes E, Rothenberg FG (2015). Cardiac troponin measurement in the critically ill: potential for guiding clinical management. J Invest Med.

[13] Salim A, Hadjizacharia P, Brown C, Inaba K, Teixeira PGR, Chan L, Rhee P, Demetriades D (2008). Significance of troponin elevation after severe traumatic brain injury. J Trauma.

[14] Bukur M, Mohseni S, Ley E, Salim A, Margulies D, Talving P, Demetriades D, Inaba K (2012). Efficacy of beta-blockade after isolated blunt head injury: does race matter?. J Trauma Acute Care Surg.

[15] Schroeppel TJ, Fischer PE, Zarzaur BL, Magnotti LJ, Clement LP, Fabian TC, Croce MA (2010). Beta-adrenergic blockade and traumatic brain injury: protective?. J Trauma.

[16] Martin M, Mullenix P, Rhee P, Belzberg H, Demetriades D, Salim A (2005). Troponin increases in the critically injured patient: mechanical trauma or physiologic stress?. J Trauma.

[17] Cai SS, Bonds BW, Hu PF, Stein DM (2016). The role of cardiac troponin I in prognostication of patients with isolated severe traumatic brain injury. J Trauma Acute Care Surg.

[18] Lippi G, Cervellin G, Lui YW (2013). Role of biomarkers in the diagnosis of mild traumatic brain injury. Radiology..

[19] El-Menyar A, Sathian B, Wahlen BM, Al-Thani H (2019). Serum cardiac troponins as prognostic markers in patients with traumatic and non-traumatic brain injuries: a meta-analysis. Am J Emerg Med.

[20] El-Menyar A, Asim M, Latifi R, Bangdiwala SI, Al-Thani H (2018). Predictive value of positive high-sensitivity troponin T in intubated traumatic brain injury patients. J Neurosurg.

[21] Komisarow J, Laskowitz DT, Mathew JP, Hernandez A, James ML, Vavilala MS, et al. TRACK-TBI Investigators. Incidence and clinical impact of myocardial injury following traumatic brain injury: a pilot TRACK-TBI study. J Neurosurg Anesthesiol. 2021;23. 10.1097/ANA.0000000000000772. 10.1097/ANA.0000000000000772 PMC8536798 33901061

[22] Bender M, Stein M, Schoof B, Kolodziej MA, Uhl E, Schöller K (2020). Troponin I as an early biomarker of cardiopulmonary parameters during the first 24 h of intensive care unit treatment in isolated traumatic brain injury patients. Injury..

[23] Mohammad Ismail A, Borg T, Sjolin G (2020). β-adrenergic blockade is associated with a reduced risk of 90-day mortality after surgery for hip fractures. Trauma Surg Acute Care Open.

[24] Chen Z, Tang L, Xu X, Wei X, Wen L, Xie Q (2017). Therapeutic effect of beta-blocker in patients with traumatic brain injury: a systematic review and meta-analysis. J Crit Care.

[25] Ko A, Harada MY, Barmparas G, Thomsen GM, Alban RF, Bloom MB, Chung R, Melo N, Margulies DR, Ley EJ (2016). Early propranolol after traumatic brain injury is associated with lower mortality. J Trauma Acute Care Surg.

[26] Khalili H, Ahl R, Paydar S, Sjolin G, Cao Y, Abdolrahimzadeh Fard H, Niakan A, Hanna K, Joseph B, Mohseni S (2020). Beta-blocker therapy in severe traumatic brain injury: a prospective randomized controlled trial. World J Surg.

[27] Murry JS, Hoang DM, Barmparas G, Harada MY, Bukur M, Bloom MB, Inaba K, Margulies DR, Salim A, Ley EJ (2016). Prospective evaluation of early propranolol after traumatic brain injury. J Surg Res.

[28] Alali AS, McCredie VA, Golan E, Shah PS, Nathens AB (2014). Beta blockers for acute traumatic brain injury: a systematic review and meta-analysis. Neurocrit Care.

[29] Bible LE, Pasupuleti LV, AlzateWD GAV, Song KJ, Sifri ZC, Livingston DH, Mohr AM (2014). Early Propranolol administration to severely injured patients can improve bone marrow dysfunction. J Trauma Acute Care Surg.

[30] Alali AS, Mukherjee K, McCredie VA (2017). Beta-blockers and traumatic brain injury: a systematic review, meta-analysis, and eastern association for the surgery of trauma guideline. Ann Surg.

[31] Neil-Dwyer G, Bartlett J, McAinsh J, Cruickshank JM (1981). Beta-adrenoceptor blockers and the blood-brain barrier. Br J Clin Pharmacol.

[32] https://www.medicines.org.uk/emc/product/2903/smpc#gref. Accessed on 25 Sept 2021

[33] Bhatia PM, Daniels LB (2020). Highly sensitive cardiac troponins: the evidence behind sex-specific cutoffs. J Am Heart Assoc.

[34] Poldermans D, Boersma E, Bax JJ, Thomson IR, van de Ven LLM, Blankensteijn JD, Baars HF, Yo TI, Trocino G, Vigna C, Roelandt JRTC, Fioretti PM, Paelinck B, van Urk H (1999). The effect of bisoprolol on peri-operative mortality and myocardial infarction in high-risk patients undergoing vascular surgery. NEJM..

[35] Ding H, Liao L, Zheng X, Wang Q, Liu Z, Xu G, Li X, Liu L (2021). β-Blockers for traumatic brain injury: a systematic review and meta-analysis. J Trauma Acute Care Surg.

[36] Cotton BA, Snodgrass KB, Fleming SB, Carpenter RO, Kemp CD, Arbogast PG, Morris JA (2007). Beta-blocker exposure is associated with improved survival after severe traumatic brain injury. J Trauma.

[37] Arbabi S, Campion EM, Hemmila MR, Barker M, Dimo M, Ahrns KS, Niederbichler AD, Ipaktchi K, Wahl WL (2007). Beta-blocker use is associated with improved outcomes in adult trauma patients. J Trauma.

[38] Inaba K, Teixeira PGR, David J-S, Chan LS, Salim A, Brown C, Browder T, Beale E, Rhee P, Demetriades D (2008). Beta-blockers in isolated blunt head injury. J Am Coll Surg.

[39] Ley EJ, Leonard SD, Barmparas G, Dhillon NK, Inaba K, Salim A (2018). et al; Beta Blockers TBI Study Group Collaborators. Beta blockers in critically ill patients with traumatic brain injury: results from a multicenter, prospective, observational American Association for the Surgery of Trauma study. J Trauma Acute Care Surg.

[40] El-Menyar A, Goyal A, Latifi R, Al-Thani H, Frishman W (2017). Brain-heart interactions in traumatic brain injury. Cardiol Rev.

[41] Patel MB, McKenna JW, Alvarez JM, Sugiura A, Jenkins JM, Guillamondegui OD, Pandharipande PP (2012). Decreasing adrenergic or sympathetic hyperactivity after severe traumatic brain injury using propranolol and clonidine (DASH After TBI Study): study protocol for a randomized controlled trial. Trials..

